# Power-Assist Add-Ons for Older Adult Manual Wheelchair Users: Protocol for a Scoping Review

**DOI:** 10.2196/56375

**Published:** 2025-05-16

**Authors:** Oladele Atoyebi, Andrew Wister, Johanne Mattie, Gloria Gutman, Habib Chaudhury, Carolyn Sparrey, O Yvette Jones, W Ben Mortenson, Eireann O’Dea, Sogol Haji Hosseini, Jaimie Borisoff

**Affiliations:** 1 Gerontology Research Center Simon Fraser University Vancouver, BC Canada; 2 Department of Gerontology Simon Fraser University Vancouver, BC Canada; 3 3MAKE+ Applied Research British Columbia Institute of Technology Burnaby, BC Canada; 4 Mechatronic Systems Engineering Simon Fraser University Surrey, BC Canada; 5 Department of Occupational Science and Occupational Therapy University of British Columbia Vancouver, BC Canada; 6 International Collaboration on Repair Discoveries Vancouver, BC Canada

**Keywords:** manual wheelchair, power add-ons, gerontology, aging, mobility, assistive technology, independence

## Abstract

**Background:**

Manual wheelchairs promote independence among older adult users. However, the user’s level of disability, strength, and stamina, and the environmental conditions in which the wheelchair is used, may limit the functionality of manual wheelchairs and their impact on independence and active aging. The use of power-assist add-ons may mitigate these limitations and allow an individual to age in place.

**Objective:**

We propose to conduct a scoping review of scientific and gray literature to examine the use of power-assist add-ons by older adults who use manual wheelchairs, as well as their advantages, limitations, and potential benefits in promoting independence and active aging.

**Methods:**

The review will be guided by the Preferred Reporting Items for Systematic reviews and Meta-Analyses extension for Scoping Reviews checklist, and we will use the Arksey and O’Malley scoping review methodology. The literature search will use a comprehensive approach and will be carried out in two phases: (1) a keyword and Medical Subject Headings search of electronic databases, proceedings, and symposia for relevant titles and abstracts and (2) a search of Google and Google Scholar. Articles will be selected based on predefined inclusion and exclusion criteria. We will include studies of power-assist add-ons for older manual wheelchair users (aged ≥65 years) across any health condition or setting. We will exclude studies not focused on power-assist add-ons or older adults. Data will be synthesized through thematic analysis (attitudes and efficacy) and directed content analysis (limitations and gaps). Results will be summarized narratively and in tables. The findings will be disseminated in peer-reviewed publications and conferences focused on rehabilitation or gerontology.

**Results:**

We will synthesize data on power-assist add-ons by compiling a list of devices, analyzing attitudes toward their use, identifying limitations, and highlighting gaps in the literature. We expect to publish the results in 2025.

**Conclusions:**

The information obtained through this review could inform future research involving wheelchair producers, suppliers, and prescribers.

**International Registered Report Identifier (IRRID):**

PRR1-10.2196/56375

## Introduction

Manual wheelchairs support the mobility of older adult users and facilitate activities of daily living, thereby promoting independence, active aging, and aging in place [1]. However, cognitive, physical, and environmental deficiencies may hinder the independence of wheelchair users, jeopardizing their ability to retain their choices to continue to live in their preferred residence and communities [2-4]. Topographic barriers may impede the free movement of wheelchair users [5]; for instance, it can be difficult to self-propel a manual wheelchair uphill or on uneven terrain [6]. There are also a variety of mental and physical limitations that may limit a person’s ability to use a manual wheelchair. For example, self-propelling a manual wheelchair requires a minimal level of coordination and upper limb strength, both of which may be absent in the user depending on their diagnosis [7]. The environmental context (eg, wheelchair accessibility, uneven or challenging terrain, and distance) poses additional constraints on manual wheelchairs [1,5,8]. All of these limitations can be exacerbated among older adults, given the normal aging process [9].

One recent development in wheelchair design that addresses these issues is power-assist add-ons for manual wheelchairs, which are gaining popularity among those who need intermittent mobility assistance (eg, for travelling longer distances or overcoming specific environmental features). There are three types of add-ons: powered front-end attachments, powered rear wheels, and powered back-end attachments. The costs of motorized wheelchairs can be prohibitive, and a power add-on for a manual wheelchair may offer a more affordable option for motorized propulsion of a manual wheelchair. A user may also prefer to enhance a manual wheelchair with which they are familiar rather than being forced to obtain and adapt to a new wheelchair, and they may prefer to switch between power and manual as desired.

Despite advances in wheelchair designs, it is not clear how power add-ons for manual wheelchairs can be deployed safely and effectively among older adults. A preliminary search of the literature revealed scoping reviews on the use of manual wheelchairs for older adults [10-12]; however, we were unable to find reviews on power-assist add-on for older adult wheelchair users, nor any registered protocol for a similar scoping review on online protocol registration platforms.

Our aim is to conduct a scoping review of scientific and gray literature in order to explore the use of power-assist add-ons by older adult users of manual wheelchairs. Our specific objectives are to (1) describe the power-assist add-ons used by older adults, if any; (2) review research studies focusing on attitudes, uptake, efficacy, and/or effectiveness of power-assist add-ons; (3) examine the limitations of power-assist add-ons among older wheelchair users; and (4) identify the gaps in existing literature. The findings of this review could inform future research involving wheelchair producers, suppliers, and prescribers.

Our research questions are as follows:

1. What power-assist add-ons exist and are used by older manual wheelchair users?
2. What are the attitudes toward uptake, efficacy, and/or effectiveness of power-assist add-ons used by older adults?
3. What are the limitations of using power-assist add-ons among older adults?
4. What are the gaps in research on the use of power-assist add-ons by older wheelchair users?

## Methods

### Protocol Registration and Study Design

Our review protocol has been registered on the Open Science Framework [13] and will be guided by the Preferred Reporting Items for Systematic reviews and Meta-Analyses Extension for Scoping Reviews (PRISMA-ScR) checklist extension for Scoping Reviews [14]. We will use the scoping review framework originally developed by Arksey and O’Malley [15] and incorporate additional refinements recommended by Levac et al [16]. This process will include the following five steps: (1) identify the research questions; (2) identify relevant studies; (3) select studies; (4) chart the data; and (5) collate, summarize, and report the results. An optional sixth stage involving consultation with stakeholders may be included, as suggested by Levac et al [16].

The PRISMA-ScR is a guideline that provides a framework for reporting the methodology and results of a scoping review. Using the elements of PRISMA-ScR can help ensure transparency and improve the reporting quality of our scoping review. We will adapt the PRISMA-ScR guidelines to fit the specific objectives and design of the scoping review as this will be helpful in ensuring transparency and completeness in reporting the review process. Some of the elements of the PRISMA-ScR checklist that we will use to provide a clear and structured presentation of the review methodology are as follows:

1. Title—clearly state that it is a scoping review.
2. Abstract—summarize the purpose, methods, and main findings of the scoping review.
3. Introduction—provide background information and rationale for the review.
4. Methods—describe the search strategy, inclusion and exclusion criteria, data sources, and data extraction process.
5. Results—present a summary of the included studies, including key characteristics and themes identified.
6. Discussion—interpret the findings, identify gaps in the literature, and discuss implications for practice and future research.
7. Conclusion—summarize the main findings and implications of the scoping review.

### Eligibility Criteria

We will include all articles that describe, assess, or explore the use of power-assist add-ons by older manual wheelchair users. Our definition of a power-assist add-on is a motorized device that can be attached to provide external power to a manual wheelchair. Our working definition of older adults will be anyone aged 65 years or older [17-19]. Based on a preliminary search of databases, we anticipate a dearth of literature focused solely on older adult wheelchair users. Hence, we will broaden the inclusion criteria to include studies done with adults and account for this approach in the discussion of our findings.

We will include older adults with all health conditions across any setting (eg, hospital, community, or rehabilitation facility). We will include the academic field of the studies, the general health-promotion programs the studies use, and the health care interventions designed for older adults with disabilities that the studies investigate.

### Search Strategy

Our literature search will use a broad approach and will be carried out in two phases.

#### Phase 1: Search up to December 2024

In phase 1, we will conduct a search of electronic databases (Multimedia Appendix 1), proceedings, and symposia for relevant titles and abstracts using a set of keywords. The keywords are generated from suggestions from team members; mining with Ovid Search Builder, Ovid Citation Analyzer, Medline, PubMed PubReMiner; and through mining within texts.

We have identified the main concepts in this scoping review and generated a list of keywords for each concept. For power-assist add-ons, we will use *power-assisted mobility devices*, *electric wheelchair attachments*, *motorized wheelchair accessories*, and *wheelchair propulsion assistance*. For manual wheelchair users, we will use *wheelchair users*, *individuals with mobility limitations*, and *people who use manual wheelchairs*. For active aging, we will use *healthy aging*, *aging well*, *independent living in older adults*, and *maintaining mobility in older adults*.

We will combine the keywords and synonyms using Boolean operators (AND, OR), as follows: (*power assist add-ons* OR *power assisted mobility devices* OR *electric wheelchair attachments* OR *motorized wheelchair accessories* OR *wheelchair propulsion assistance*) AND (*manual wheelchair users* OR *wheelchair users* OR *individuals with mobility limitations* OR *people who use manual wheelchairs*) AND (*aging* OR *healthy aging* OR *aging well* *OR independent living in older adults* OR *maintaining mobility in older adults*).

Truncation or wildcard symbols (*) will be used to capture variations of keywords: (*power assist add-ons* OR power assist* OR electric wheelchair attach* OR motorized wheelchair access* OR wheelchair propulsion assist*) AND (*manual wheelchair users* OR wheelchair user* OR individual* with mobility limitation* OR people who use manual wheelchair*) AND (*aging* OR *healthy aging* OR *aging well* OR independent living in older adult* OR maintain* mobility in older adult*).

In addition, Medical Subject Headings (MeSH) and free-text search terms will be applied to each database without limitations for publication period. We will use relevant MeSH terms, such as *wheelchairs* OR *assistive technology* OR *power assisted mobility devices* OR *manual wheelchair users* OR *power add-on* OR *powered rear wheels* OR *back-end attachment* OR *front-end attachment* OR *aged* OR *aging* OR *mobility limitations* OR *rehabilitation* OR *physical therapy* OR *occupational therapy* OR *assistive devices* OR *mobility aids* OR *accessibility* OR *independent living* OR *quality of life* OR *user satisfaction* OR *health outcomes* OR *functionality* OR *ergonomics* OR *safety*.

#### Phase 2: Search up to First 10 Result Pages

In phase 2, we will search Google and Google Scholar using the same keywords applied in the first phase. Additional relevant articles will be extracted from reference lists of similar reviews and papers. All articles resulting from our search will then be uploaded to the online platform Covidence to eliminate duplicates and for subsequent screening. An example of the search strategy for the Ovid database is presented in Multimedia Appendix 2.

### Study Selection

Before we carry out the formal screening, a calibration exercise (pilot testing in 10% of identified studies) will be completed to ensure reliability. Percentage agreement will need to be >80% across reviewers to begin formal screening. Training, screening, and further development of criteria will occur until this interrater agreement threshold is met. The screening will be performed independently by 2 review authors, and discrepancies will be resolved by consensus or with the involvement of a third reviewer. The screening of articles will be completed in two stages. In the first stage, 2 researchers will independently screen the titles and abstracts, and conflicts will be resolved through dialogue or by a third researcher. In the second stage, full-text versions of the resulting articles will be obtained and reviewed by the 2 researchers. Articles will be selected based on the inclusion and exclusion criteria.

### Data Charting

Data charting has four objectives: (1) we will enumerate the kinds of power-assist add-ons identified in the review, and we will create a list of names of all add-ons; (2) we will use thematic analysis to explore and identify themes and summarize attitudes toward uptake, efficacy, and/or effectiveness of power-assist add-ons for older adult wheelchair users [20,21]; (3) for any data on the limitations of power-assist add-ons, we will apply directed content analysis to describe the challenges associated with their use, areas for improvement, and strategies to improve quality, application, usability, and acceptance [22]; and (4) in aspects of this review where there is a paucity of data to answer our research question or where some specific methodologies or content are lacking, we will identify these as gaps.

### Data Items

For each of the included studies, we will extract the following data: study characteristics, including the study’s year and setting (hospital, rehabilitation facility, community, or other); type of add-on; type of health care provider (if applicable); condition studied or age group (as applicable); country or region of origin; funded vs unfunded status; and journal name. We will also record the study’s purpose and objectives, including its aim, questions, topic (or concept) addressed, and outcomes (ie, identified evidence gaps, future research opportunities, strengths and limitations, and implications for policy or practice), as well as the study’s methodology, approach, and scope.

### Synthesis of Results

The synthesis of this scoping review will focus on 3 key aspects: the uptake, effectiveness, and limitations of power-assist add-ons for older manual wheelchair users. Thematic analysis will be used to identify and organize key themes from the data, ensuring that the findings reflect a comprehensive understanding of users’ attitudes toward these devices.

Themes will emerge through an inductive, iterative process, with data coded and grouped based on similarities and patterns. Each theme will explore factors such as the perceived benefits, challenges, and barriers to adopting power-assist add-ons. The synthesis will also examine how factors like physical, emotional, and social considerations influence users’ experiences.

By ensuring methodological rigor and reflexivity, the synthesis will provide a clear, cohesive narrative of older adults’ experiences with power-assist add-ons, highlighting critical factors for adoption and their overall effectiveness. The final synthesis will contribute to a deeper understanding of this technology’s role in improving mobility for older wheelchair users.

### Dissemination

Dissemination of our findings will include (1) producing versions of an executive summary that will be tailored to various groups, including educators, prescribers, and developers, as we expect that our findings will inform further collaboration with clinicians, prescribers, and dealers; (2) publishing in peer-reviewed journals focused on in rehabilitation and/or gerontology and on online platforms; and (3) presenting at relevant conferences (eg, the conference of the Canadian Association on Gerontology).

## Results

We expect this scoping review to provide a comprehensive overview of the types of power-assist add-ons used by older adult manual wheelchair users. This could include motorized rear-wheel attachments and other propulsion devices. We anticipate identifying a wide range of power-assist technologies, along with variations in their adoption, usability, and perceived efficacy across different settings and populations.

We expect to discover a wide range of attitudes regarding the adoption and effectiveness of these devices. We expect to find that some users report notable improvements in mobility and independence and a reduction in physical strain. However, challenges such as cost, maintenance difficulties, and concerns related to the device’s weight and comfort may also emerge as key factors affecting adoption and use.

## Discussion

### Anticipated Findings

We foresee identifying several limitations related to the use of power-assist add-ons, including challenges with device integration, safety concerns, and insufficient training or support for users. These limitations are likely to be discussed in the context of how power-assist technologies are implemented in various settings, as well as the potential gaps in existing support systems.

Additionally, we expect this review to reveal mixed perceptions regarding the overall impact of power-assist add-ons on the quality of life and daily functioning of older adults. While some users may experience considerable improvements in these areas, others may face difficulties with the practicality and long-term sustainability of the devices.

We also expect that this scoping review will highlight gaps in the literature, particularly in the areas of long-term outcomes, cost-effectiveness, and the inclusion of older adults with diverse physical abilities and needs. This will help pinpoint areas requiring further research and development. Finally, the findings will inform recommendations for future research aimed at improving the design, usability, and accessibility of power-assist add-ons for older adults, with a focus on addressing identified barriers and enhancing user experience.

### Conclusion

It is well known that older adults should be provided the opportunity to maximize independence, social connections, and quality of life. For older manual wheelchair users, these conditions for successful aging could potentially be enhanced by power-assist add-ons. We anticipate that the proposed review will summarize and provide key information to support future research and to enhance the knowledge base needed to further improve the use of power-assist add-ons.

## Appendix

Multimedia Appendix 1 List of databases.

Multimedia Appendix 2 Ovid search sample.

Multimedia Appendix 3 Preferred Reporting Items for Systematic reviews and Meta-Analyses extension for Scoping Reviews (PRISMA-ScR) Checklist.

## Abbreviations

MeSH: Medical Subject Headings
PRISMA-ScR: Preferred Reporting Items for Systematic reviews and Meta-Analyses extension for Scoping Reviews

## References

[1] Bigonnesse C, Mahmood A, Chaudhury H, Mortenson WB, Miller WC, Martin Ginis KA (2018). The role of neighborhood physical environment on mobility and social participation among people using mobility assistive technology. Disabil Soc.

[2] Engineer A, Sternberg E, Najafi B (2018). Designing interiors to mitigate physical and cognitive deficits related to aging and to promote longevity in older adults: a review. Gerontology.

[3] How T, Wang RH, Mihailidis A (2013). Evaluation of an intelligent wheelchair system for older adults with cognitive impairments. J Neuroeng Rehabil.

[4] Noreau Luc, Boucher Normand, Edwards Geoffrey, Fougeyrollas Patrick, Morales Ernesto, Routhier Francois, Vincent Claude, Gascon Hubert, Dietz V, Ward NS (2020). Enhancing independent community access and participation: services, technologies, and policies. Oxford Textbook of Neurorehabilitation.

[5] Menzies A, Mazan C, Borisoff JF, Mattie JL, Mortenson WB (2021). Outdoor recreation among wheeled mobility users: perceived barriers and facilitators. Disabil Rehabil Assist Technol.

[6] Khalili M, Jonathan C, Hocking N, Van der Loos M, Mortenson WB, Borisoff J (2023). Perception of autonomy among people who use wheeled mobility assistive devices: dependence on environment and contextual factors. Disabil Rehabil Assist Technol.

[7] Abou L, Rice LA (2024). The differences in demographics, fear of falling, transfer quality and participation enfranchisement between manual and power wheelchair users with multiple sclerosis and spinal cord injury. Disabil Rehabil Assist Technol.

[8] Ramriez DZM, Holloway C (2017). "But, I don't want/need a power wheelchair": toward accessible power assistance for manual wheelchairs. ASSETS '17: Proceedings of the 19th International ACM SIGACCESS Conference on Computers and Accessibility.

[9] Requejo P, Furumasu J, Mulroy S (2015). Evidence-based strategies for preserving mobility for elderly and aging manual wheelchair users. Top Geriatr Rehabil.

[10] Choukou M, Best KL, Potvin-Gilbert M, Routhier F, Lettre J, Gamache S, Borisoff JF, Gagnon D (2021). Scoping review of propelling aids for manual wheelchairs. Assist Technol.

[11] Mason B, Warner M, Briley S, Goosey-Tolfrey V, Vegter R (2020). Managing shoulder pain in manual wheelchair users: a scoping review of conservative treatment interventions. Clin Rehabil.

[12] Routhier F, Lettre J, Miller WC, Borisoff JF, Keetch K, Mitchell IM, Research Team C (2018). Data logger technologies for manual wheelchairs: a scoping review. Assist Technol.

[13] Scoping review: power assist add-ons for manual wheelchairs users: a protocol for an active aging lens scoping review. Open Science Network.

[14] Tricco AC, Lillie E, Zarin W, O'Brien KK, Colquhoun H, Levac D, Moher D, Peters MD, Horsley T, Weeks L, Hempel S, Akl EA, Chang C, McGowan J, Stewart L, Hartling L, Aldcroft A, Wilson MG, Garritty C, Lewin S, Godfrey CM, Macdonald MT, Langlois EV, Soares-Weiser K, Moriarty J, Clifford T, Tunçalp Özge, Straus SE (2018). PRISMA Extension for Scoping Reviews (PRISMA-ScR): checklist and explanation. Ann Intern Med.

[15] Arksey H, O'Malley L (2005). Scoping studies: towards a methodological framework. Int J Soc Res Methodol.

[16] Levac D, Colquhoun H, O'Brien KK (2010). Scoping studies: advancing the methodology. Implement Sci.

[17] Sakakibara BM, Miller WC, Eng JJ, Backman CL, Routhier F (2013). Preliminary examination of the relation between participation and confidence in older manual wheelchair users. Arch Phys Med Rehabil.

[18] Korotchenko A, Hurd Clarke L (2013). Power mobility and the built environment: the experiences of older Canadians. Disabil Soc.

[19] Turcotte M, Schellenberg G (2007). A portrait of seniors in Canada. Statistics Canada.

[20] Braun V, Clarke V, Hayfield N, Terry G, Liamputtong P (2019). Thematic analysis. Handbook of Research Methods in Health Social Sciences.

[21] Nowell LS, Norris JM, White DE, Moules NJ (2017). Thematic analysis: Striving to meet the trustworthiness criteria. Int J Qual Methods.

[22] Mayring P (2000). Qualitative content analysis. Qual Soc Res.

