# Supplementary material for: Power-Assist Add-Ons for Older Adult Manual Wheelchair Users: Protocol for a Scoping Review
Source: JMIR Res Protoc. 2025 May 16;14:e56375. doi: 10.2196/56375 (PMC12125556; doi:10.2196/56375)
Supplement: Multimedia Appendix 1 [file resprot_v14i1e56375_app1.docx]

**Multimedia Appendix 3: List of databases**

- Pubmed
- Medline
- Embase
- CINAHL
- Compendex
- IEEE Xplore
- Rehabilitation Engineering Society of America (RESNA) proceedings
- International Seating Symposium (ISS) proceedings
- European Seating Symposium (ESS) proceedings
- RESNA and ISS proceedings
- Ageline
- Psychinfo
- PsycArticles
- Environment complete
- Ergonomics abstracts
- Global health
- Ovid MEDLINE/PubMed,
- BMJ Open
- Cochrane Database of Systematic Reviews
- Joanna Briggs Institute (JBI) EBP database
- JBI Evidence Synthesis
- JBI Evidence Implementation
- The JBI Database of Systematic Reviews and Implementation Reports
- Google, and Google Scholar
