# Supplementary material for: Power-Assist Add-Ons for Older Adult Manual Wheelchair Users: Protocol for a Scoping Review
Source: JMIR Res Protoc. 2025 May 16;14:e56375. doi: 10.2196/56375 (PMC12125556; doi:10.2196/56375)
Supplement: Multimedia Appendix 2 [file resprot_v14i1e56375_app2.docx]

**Multimedia Appendix 4: Ovid search sample**

(add-on* OR add on* OR power wheel* OR PAPAW OR power assist OR electric assist OR wheelchair mobility aid* OR attachments OR e-motion OR batec OR twin OR smart drive OR Propelling aid*

AND

wheelchair* OR manual wheelchair OR pushrim activated wheelchair)

AND

(older adult* OR seniors OR elderly OR elder)

AND

(mobility OR active aging OR aging in place OR activities of daily living OR independence OR community participation OR quality of life OR usability OR ADL)

[(Engineer et al., 2018)](https://ovidsp-dc2-ovid-com.proxy.lib.sfu.ca/ovid-a/ovidweb.cgi?&S=LCOJFPHDPCEBNIEHIPMJOGLEIMFIAA00&Database+Field+Guide=13)**EBM Reviews - Cochrane Database of Systematic Reviews**2005 to December 1, 2022**,**[[
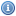
](https://ovidsp-dc2-ovid-com.proxy.lib.sfu.ca/ovid-a/ovidweb.cgi?&S=LCOJFPHDPCEBNIEHIPMJOGLEIMFIAA00&Database+Field+Guide=11)](https://ovidsp-dc2-ovid-com.proxy.lib.sfu.ca/ovid-a/ovidweb.cgi?&S=LCOJFPHDPCEBNIEHIPMJOGLEIMFIAA00&Database+Field+Guide=11)**EBM Reviews - ACP Journal Club**1991 to November 2022**,**[[
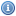
](https://ovidsp-dc2-ovid-com.proxy.lib.sfu.ca/ovid-a/ovidweb.cgi?&S=LCOJFPHDPCEBNIEHIPMJOGLEIMFIAA00&Database+Field+Guide=14)](https://ovidsp-dc2-ovid-com.proxy.lib.sfu.ca/ovid-a/ovidweb.cgi?&S=LCOJFPHDPCEBNIEHIPMJOGLEIMFIAA00&Database+Field+Guide=14)**EBM Reviews - Cochrane Clinical Answers**November 2022**,**[[
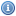
](https://ovidsp-dc2-ovid-com.proxy.lib.sfu.ca/ovid-a/ovidweb.cgi?&S=LCOJFPHDPCEBNIEHIPMJOGLEIMFIAA00&Database+Field+Guide=3)](https://ovidsp-dc2-ovid-com.proxy.lib.sfu.ca/ovid-a/ovidweb.cgi?&S=LCOJFPHDPCEBNIEHIPMJOGLEIMFIAA00&Database+Field+Guide=3)**EBM Reviews - Database of Abstracts of Reviews of Effects**1st Quarter 2016**,**[[
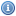
](https://ovidsp-dc2-ovid-com.proxy.lib.sfu.ca/ovid-a/ovidweb.cgi?&S=LCOJFPHDPCEBNIEHIPMJOGLEIMFIAA00&Database+Field+Guide=15)](https://ovidsp-dc2-ovid-com.proxy.lib.sfu.ca/ovid-a/ovidweb.cgi?&S=LCOJFPHDPCEBNIEHIPMJOGLEIMFIAA00&Database+Field+Guide=15)**Ovid MEDLINE(R) and Epub Ahead of Print, In-Process, In-Data-Review & Other Non-Indexed Citations, Daily and Versions**1946 to December 1, 2022
